# Mouse ECG findings in aging, with conduction system affecting drugs and in cardiac pathologies: Development and validation of ECG analysis algorithm in mice

**DOI:** 10.14814/phy2.12639

**Published:** 2015-12-10

**Authors:** Mari Merentie, Jukka A. Lipponen, Marja Hedman, Antti Hedman, Juha Hartikainen, Jenni Huusko, Line Lottonen‐Raikaslehto, Viktor Parviainen, Svetlana Laidinen, Pasi A. Karjalainen, Seppo Ylä‐Herttuala

**Affiliations:** ^1^ Department of Biotechnology and Molecular MedicineA. I. Virtanen Institute for Molecular SciencesFaculty of Health SciencesUniversity of Eastern FinlandKuopioFinland; ^2^ Department of Applied PhysicsUniversity of Eastern FinlandKuopioFinland; ^3^ Heart CenterKuopio University HospitalKuopioFinland; ^4^ Diagnostic Imaging CenterKuopio University HospitalKuopioFinland; ^5^ Science Service CenterKuopio University HospitalKuopioFinland; ^6^ Gene Therapy UnitKuopio University HospitalKuopioFinland

**Keywords:** Aging, left ventricular hypertrophy, mouse ECG, myocardial infarction

## Abstract

Mouse models are extremely important in studying cardiac pathologies and related electrophysiology, but very few mouse ECG analysis programs are readily available. Therefore, a mouse ECG analysis algorithm was developed and validated. Surface ECG (lead II) was acquired during transthoracic echocardiography from C57Bl/6J mice under isoflurane anesthesia. The effect of aging was studied in young (2–3 months), middle‐aged (14 months) and old (20–24 months) mice. The ECG changes associated with pharmacological interventions and common cardiac pathologies, that is, acute myocardial infarction (AMI) and progressive left ventricular hypertrophy (LVH), were studied. The ECG raw data were analyzed with an in‐house ECG analysis program, modified specially for mouse ECG. Aging led to increases in P‐wave duration, atrioventricular conduction time (PQ interval), and intraventricular conduction time (QRS complex width), while the R‐wave amplitude decreased. In addition, the prevalence of arrhythmias increased during aging. Anticholinergic atropine shortened PQ time, and beta blocker metoprolol and calcium‐channel blocker verapamil increased PQ interval and decreased heart rate. The ECG changes after AMI included early JT elevation, development of Q waves, decreased R‐wave amplitude, and later changes in JT/T segment. In progressive LVH model, QRS complex width was increased at 2 and especially 4 weeks timepoint, and also repolarization abnormalities were seen. Aging, drugs, AMI, and LVH led to similar ECG changes in mice as seen in humans, which could be reliably detected with this new algorithm. The developed method will be very useful for studies on cardiovascular diseases in mice.

## Introduction

ECG is the cornerstone in diagnosing patients with heart disease. Mice have become increasingly important in studying cardiac diseases mainly because of the growing number of genetically modified strains (Scherrer‐Crosbie and Thibault 2008; Boukens et al. 2014). However, very little is known about the mouse ECG and its relation to physiological and pathological changes in the heart. Therefore, accurate ECG analysis systems for mouse ECG are needed.

Mouse ECG is known to be somewhat different compared with human. Both in mouse and human ECG, the atrial depolarization is seen as a P wave. It is followed by PQ interval which reflects the conduction time from atria via AV node and His‐Purkinje system to ventricles, after which ventricular depolarization is seen as the QRS complex (Kaese and Verheule 2012; Boukens et al. 2014). Due to differences in ventricular action potential shape between the two species, the mouse ECG has so‐called J wave at the end of QRS complex, which takes place in the beginning of repolarization, and there is no isoelectric ST segment (Boukens et al. 2013; Speerschneider and Thomsen 2013). In mouse cardiac chambers, the repolarization happens gradually, and there is no clear separate positive T wave as seen in humans, but the negative T wave merges with the final part of the QRS complex. It is thought that the QT interval ends when the ECG deflection returns to the isoelectric line after QRS complex and J wave (Kaese and Verheule 2012; Boukens et al. 2014). In addition, differences in the ECG between different inbred mouse strains have been reported (Wehrens et al. 2000; Kaese and Verheule 2012).

To our knowledge, the effect of aging on ECG in C57Bl/6J mice has not been previously reported. In 24‐month‐old Kunming mice, the P‐wave duration is increased compared with 2‐month‐old controls (Luo et al. 2013) and the PR interval increases with aging in 129S1 female mice (Xing et al. 2009). ECG changes in common cardiac diseases, acute myocardial infarction (AMI), and left ventricular hypertrophy (LVH) have not been widely studied in mice. With FVB strain, it was shown that the ST elevation is seen 1 h and the development of pathological Q waves 4 and 14 days after the AMI (Gehrmann et al. 2001), which resembles changes seen in human AMI (Thygesen et al. 2012). Surface ECG changes in the compensatory phase of the progressive LVH in the widely used transversal aortic constriction (TAC) induced disease model have not been studied in mice. In human LVH, the QRS complex amplitude is increased in certain ECG leads, QRS complex is usually wider, and there might be secondary repolarization abnormalities (Thaler 2010).

Accurate, convenient, and noninvasive measurement of ECG in mice is critical for characterizing several disease phenotypes and translating these findings to human diseases. Therefore, we developed an ECG analysis algorithm for mice and validated it with aging, medication, and under certain pathological conditions. The ECG was recorded simultaneously with the echocardiography allowing easy comparison with the echocardiographic findings. The analyses produced reliable information aiding the study of human cardiac diseases in mouse models.

## Materials and Methods

### Experimental animals

In total, 269 C57Bl/6J male mice (Harlan Laboratories, Indianapolis, IN) were used for the experiments. All of the animal procedures were approved by The National Animal Experiment Board of Finland and carried out in accordance with the guidelines of The Finnish Act on Animal Experimentation. The animals were kept in standard housing conditions in The National Laboratory Animal Center of The University of Eastern Finland, Kuopio. Diet and water were provided ad libitum.

### The effects of aging and selected drugs

The changes caused by aging were studied with three C57Bl/6J mice groups of different age: 2–3 months old (young, *n* = 73), 12–14 months old (middle aged, *n* = 34), and 20–24 months old (old, *n* = 40) mice. After sacrification, hearts from young and old mice were collected and processed for histology. Hearts were perfused with 1% paraformaldehyde (PFA) solution and immersion fixed with 4% PFA in 7.5% sucrose for 4 h and kept in 15% sucrose overnight. Hematoxylin–eosin (HE) and Masson trichrome (Accustain trichrome stains; Sigma‐Aldrich, St Louis, MO) stainings were done from 5‐*μ*m‐thick paraffin‐embedded sections and used for studying the general tissue morphology and the size of the infarction scar.

The effects of conduction system affecting drugs, 2 mg/kg atropine (ATROPIN 1 mg/ml, Takeda Oy, Helsinki, Finland), 2 mg/kg metoprolol (SELOKEN, AstraZeneca, Espoo, Finland), and 5 mg/kg verapamil (VERPAMIL, Orion, Espoo, Finland) were studied in 5‐month‐old C57BL/6J mice. Drugs were given with intraperitoneal (ip) injections, and saline (0.9% NaCl) injections were used as a control, *n* = 6/group, except *n* = 4 in atropine group. The used dilutions were adjusted to equalize the injected volume with all the drugs and saline (10 *μ*L/g mouse weight). Baseline measurements were done prior to injections, and the effects of drugs were monitored with ECG recorded during transthoracic echocardiography 5, 10, 15, and 20 min after the administration of the drug the mice being under isoflurane anesthesia throughout the follow‐up.

### Myocardial infarction model and model for progressive LVH

The effect of myocardial ischemia was studied in 3‐month‐old C57Bl/6J mice by using a novel less invasive surgical model of AMI described previously (Gao et al. 2010). Briefly, the heart was gently “popped out” through a small incision made at the fourth intercostal space, and the left anterior descending (LAD) coronary artery was sutured and ligated using a 6–0 silk suture at a site less than 5 mm from its origin. After the ligation, the heart was immediately put back into the intrathoracic cavity and the muscle layers and skin were closed. The same surgical procedure was done for the sham‐operated group without the LAD ligation: *n* = 6 in MI group and *n* = 4 in sham group. Each MI was confirmed by echocardiographic visualization and histological findings. Echocardiography and ECG were recorded prior to the operation (baseline), 1, 4, and 8 h and 1, 5, and 21 days (d) post operation. The mice were sacrificed 21 days after the operation, and hearts were collected and processed for histology as with the mice of the aging study.

Progressive LVH was induced by chronic pressure overload as a result of TAC operation as described previously (Huusko et al. 2012) for 3‐ to 4‐month‐old mice (*n* = 8–10/group). ECG was recorded 2 and 4 weeks after the TAC and sham operations.

### ECG recording during transthoracic echocardiography

Surface ECG signal (lead II via limb electrodes) was acquired during high‐resolution transthoracic echocardiography (TTE) with Vevo 2100 Ultrasound System designed for small animals (Fujifilm VisualSonics Inc., Toronto, Ontario, Canada). A MS‐400 high‐frequency ultrasound probe operating at 18–38 MHz was used. The mice were placed in supine position on a heated platform (THM100; Indus Instruments, Houston, TX) in order to maintain their body temperature at 36–37°C, which was monitored via a rectal probe. Prior to the ECG recordings, the mice were acclimatized to the anesthesia for 10–15 min, during which they were prepared for the simultaneous echocardiography. For recording the ECG signal representing the standard limb lead II, the paws of the mice were connected to the platform's electrode pads with the aid of ECG gel and fixed with a skin tape. Also, heart rate (HR) and respiration were monitored during anesthesia via the ECG pads. The hair from the chest was carefully removed with a depilatory (Veet, Reckitt Benckiser, UK).

### Electrocardiography analysis

The Electrocardiography (ECG) data were exported from the Vevo software as raw data format, and digital signal processing was performed with in‐house software written in the Matlab (MathWorks, Natick, MA). The software was developed with experts in cardiology and electrophysiology specially for analyzing mouse ECG by taking into account the specific features of the mouse ECG. Sixty seconds of ECG signal were recorded at a sampling rate of 8000 Hz, and 30‐sec time interval having the smallest variation of the HR was chosen for the final analysis. The whole recorded ECG tracing was visually reviewed for detecting possible arrhythmias or other aberrant ECG complexes. R‐peak fiducial points were detected using automatic QRS‐detection algorithm (Kubios HRV; Tarvainen et al. 2014); in addition, all detections were visually verified. Averaged PQRST wave epoch was calculated using [−0.1 to 0.1] sec time window with R peaks as the trigger point (zero point). From the averaged wave epoch, onset and offset of the each ECG wave were determined automatically using first derivative threshold method (for more details, see, e.g., Vazquez‐Seisdedos et al. 2011). However, wave positions were always verified and corrected by the trained specialist. Defined timepoints were onset and offset of the P‐wave, Q‐wave onset, and R‐ and S‐wave peaks and offset of the QRS, QRSp and T wave. By using these timepoints P‐wave duration, PQ interval, Q wave duration, QRS and QRSp width, and QT interval were estimated. In addition, amplitudes of the P, Q, R, and S waves were defined using onset of the P wave as isoelectric line.

P‐wave duration as a measure of intra‐atrial conduction was measured from the onset of the P wave to the point where the P wave returns to the baseline; the PQ interval representing intra‐atrial and atrioventricular conduction from the beginning of the P wave to the beginning of the QRS complex; QRS complex representing ventricular depolarization from the beginning of the Q or R wave until the temporal halfway of S‐wave peak and J‐wave peak; J wave, the additional wave in the early repolarization right after the QRS complex (Liu et al. 2004); QRSp, time interval developed herein, representing ventricular depolarization and early repolarization, the time from the beginning of the QRS complex to the end of the J wave, which is of the point where J wave returns to the isoelectric line; QT time represents the duration of ventricular depolarization and repolarization and is the time between the start of the QRS complex and the end of the T wave, when the curve returns to the isoelectric line or to the point where the first derivative of T‐wave end became nearly zero; QTc, QT time corrected for RR interval with the formula specifically modified for mice from the Bazett's formula used in human ECG analysis: QTc = mean QT/(RR/100)^1/2^ (the unit for QT and RR is msec) (Mitchell et al. 1998). Arrhythmias were detected from the ECG tracings as premature atrial or ventricular complexes, irregular RR intervals equivalent to atrial fibrillation, or wide complex tachycardia.

### Transthoracic echocardiography measurements

Along the ECG recordings, also TTE parameters were acquired. The left ventricle (LV) dimensions and ejection fraction (EF) were determined from parasternal short‐axis M‐mode images acquired at the midpapillary level of LV. The Vevo software calculated EF with the Teichholz formula and LV Mass with the following formula: 1.053 × [(LVEDD;d + LVPW;d + LVAW;d)^3^ − LVEDD^3^] (LVEDD, left ventricle end‐diastolic diameter; LVPW, LV posterior wall; LVAW, LV anterior wall). The left atrium (LA) area was determined with the 2D area tool from parasternal long‐axis view B‐Mode image taken more laterally than the normal long‐axis view to visualize the LA at its largest point. Also M‐Mode projection of aortic root and LA diameter were taken from the same orientation. For obtaining global LV function measurements after AMI, the LV trace mode was used to analyze the parasternal long‐axis view B‐Mode images. In the LV trace mode, the endocardial borders are traced in end‐systole and in end‐diastole and the system will then interpolate the traces between the outlines at end‐systole and end‐diastole, after which it counts the needed parameters. EF in LV trace is calculated with the following formula EF = 100 * stroke volume/diastolic volume (stroke volume = diastolic volume‐systolic volume, where diastolic/systolic volume is V d/s = 7.0/(2.4 + average systolic/diastolic diameter) * (average systolic/diastolic diameter)).

### Statistical analyses

Statistical analyses were performed with Excel software with Student's paired *t*‐test when comparing two groups and with GraphPad Prism 6.0 software (GraphPad Software, Inc., La Jolla, CA) using one‐way analysis of variance with Dunnett's post hoc test when comparing three or more groups/timepoints. The used tests are indicated in the figure legends. *P* value <0.05 was considered statistically significant, and the following symbols were used for *P* value: *P <* *0.05, **P <* *0.01, and ***P <* *0.001. Correlations between echocardiographic and ECG measurements were studied with the GraphPad Prism 6.0 software by calculating the value of the Pearson correlation coefficient (*r*).

## Results

### ECG and echocardiographic findings during aging

**Figure 1:**
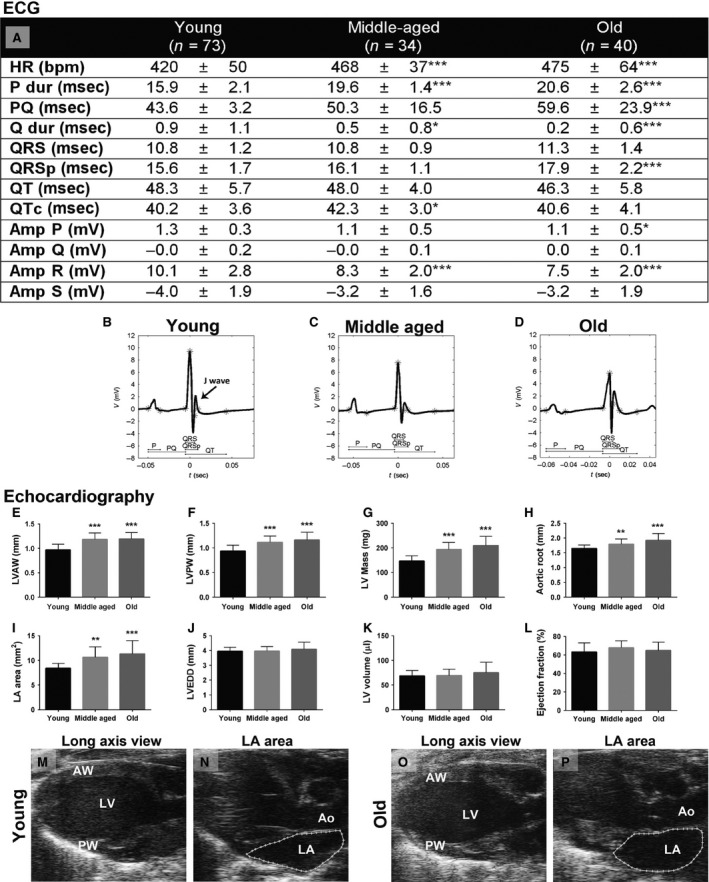
ECG and echocardiographic results of young (2–3 months old, *n* = 73), middle‐aged (12–14 months old, *n* = 34), and old (20–24 months old, *n* = 40) C57Bl/6J mice. ECG measurements of the lead II (A) and representative ECG of young (B), middle‐aged (C), and old (D) mice with appropriate ECG intervals marked. Echocardiographic measurements of LVAW thickness (E), LVPW thickness (F), LV mass (G), aortic root diameter (H), LA area (I), LVEDD (J), LV volume (K), and ejection fraction (L). Representative long‐axis view B‐Mode images of young (M, N) and old (O, P) mice. HR, heart rate; P dur, duration of P wave; Q dur, duration of Q wave; Amp, amplitude; LV, left ventricle; LVAW, LV anterior wall; LVPW, LV posterior wall; LVEDD, LV end‐diastolic diameter; LA, left atrium. Results are expressed as mean ± SD. One‐way ANOVA with Dunnett's post hoc test was used, *P < 0.05, **P < 0.01, and ***P < 0.001 compared with the group of young mice.

**Figure 2:**
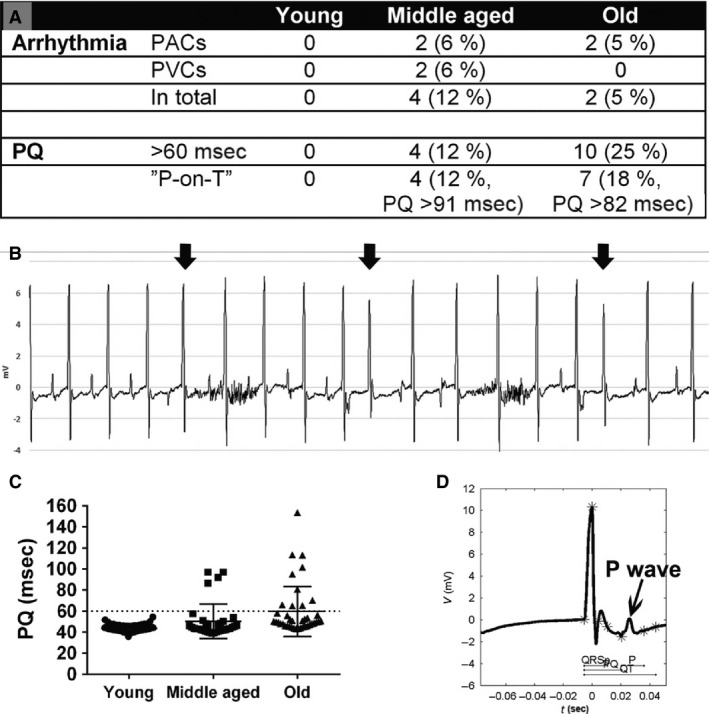
Arrhythmias and long PQ interval in aged mice. (A) The prevalence of arrhythmias, long PQ interval >60 msec, and unusually long PQ interval with “P‐on‐T” phenomenon (P wave superimposed on the T wave of the previous complex) represented as number of mice (% of mice). (B) The arrhythmias in the middle‐aged and in the old mice group were most commonly PACs (arrows) with aberrant P waves. (C) PQ interval duration in young, middle‐aged, and old mice: *n* = 73/34/40 in the groups of young/middle‐aged/old mice, respectively. PAC, premature atrial contraction; PVC, premature ventricular contraction.

The effects of aging were studied in young (2–3 months old), middle‐aged (12–14 months old), and old (20–24 months old) C57Bl/6J mice. ECG analysis results showed that HR, duration of P wave, and atrioventricular conduction interval (PQ interval) were increased in middle‐aged and old mice compared with the young mice (Fig. 1A–D, Fig. 2B). There were no changes in ventricular conduction time (QRS complex width), whereas QRSp duration was increased significantly in the group of old mice as a result of widening of the J wave. In addition, R‐wave amplitude decreased in middle‐aged and old mice. Echocardiographic results showed that LVAW, LVPW, LV mass, aortic root diameter, and LA area were increased in middle‐aged and old mice (Fig. 1E–I, M–P). Instead, there were no changes in LVEDD, LV volume, or systolic function measured by Teichholz EF (Fig. 1J–L).

All mice had sinus rhythm during the ECG recording. There were no arrhythmias detected in the group of young mice (Fig. 2A). Instead, the prevalence of arrhythmias was 12% in the middle‐aged group and 5% in the old mice group, and the arrhythmias were mostly premature atrial contractions (PACs) with aberrant P waves (Fig. 2A, B). All young mice had PQ interval below 55 msec, and therefore, PQ interval above 60 msec was thought to be clearly above the normal range. When the percentage of the mice having a PQ interval above 60 msec was counted, 12% of the middle‐aged mice and 25% of the old mice had increased PQ interval (Fig. 2A, C). Some mice in the middle‐aged and old mice groups had such a long PQ interval that the P wave was superimposed on the T wave of the previous complex and had PQ interval over 91 msec in middle‐aged group and PQ interval over 82 msec in the old mice group (Fig. 2A, C, D). There was a slight deformation of the QRS complex in 24% of the middle‐aged and 10% of the old mice.

The mice gained weight during aging as follows: weight of the young mice was 26.0 ± 3.0 g, middle‐aged mice 43.5 ± 6.2 g (***), and old mice 45.4 ± 8.0 g (***). Although LV mass was increased during aging (Fig. 1G), the LV mass/mouse weight ratio decreased as follows: young mice 5.9 ± 0.8 mg/g, middle‐aged mice 4.6 ± 0.5 mg/g (***), and old mice 4.8 ± 1.1 mg/g (***). Results are expressed as mean ± SD. ***P < 0.001 compared with the group of young mice. The general histology of the hearts of the old mice was essentially normal, and there was no detectable fibrosis (data not shown).

### Correlations of ECG and echocardiographic measurements

To study how well the ECG findings correlated with the echocardiographic findings, the results of the 2‐ to 24‐month old mice in the aging study were subjected for the correlation analysis. A significant correlation between LA size with P‐wave duration was found (Table 1). The correlation of P‐wave duration was better with LA area than with LA diameter. The size of the LV mass correlated with QRSp width and PQ interval but not with QRS complex width. No significant correlation was found between HR and PQ interval, but P‐wave duration correlated significantly with HR.

**Table 1:** Correlation analysis of echocardiographic and ECG measurements of 2‐ to 24‐month‐old C57Bl/6J mice

Echocardiography versus ECG Correlation	*n*	*r*	*P*
LA diameter versus P dur	114	0.33	0.0004***
LA area versus P dur	90	0.42	<0.0001***
LV Mass versus QRS	147	0.16	0.0606 ns
LV Mass versus QRSp	147	0.37	<0.0001***
LV Mass versus PQ	147	0.24	0.0038**
PQ versus HR	147	0.12	0.1559 ns
P dur versus HR	121	0.30	0.0009***

*n*, number of mice; *r*, correlation coefficient; *P*,* P* value of significance; LA, left atrium; P dur, duration of P wave; P Amp, Amplitude of P wave; LV, Left ventricle; HR, heart rate.

### The effects of atropine, metoprolol, verapamil, and saline on heart rate, PQ interval, and ejection fraction

**Figure 3:**
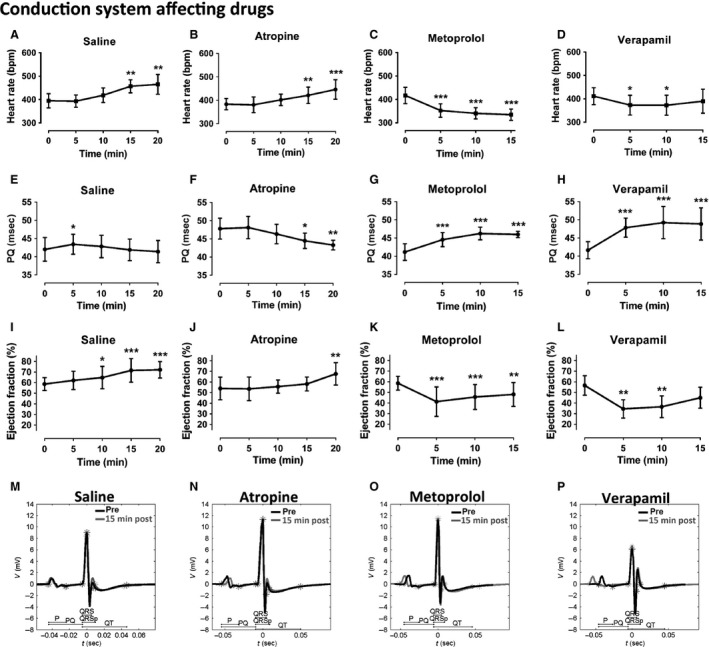
Effects of conduction system affecting drugs atropine, metoprolol, verapamil, and control saline on heart rate (A–D), PQ interval (E–H), and ejection fraction (I–L). Representative ECGs of each group before (black) and 15 min after (gray) the administration of the drug via ip injection (M–P). Results are expressed as mean ± SD. One‐way repeated‐measures ANOVA with Dunnett's post hoc test was used, *P < 0.05, **P < 0.01, ***P < 0.001 compared with the baseline (0 min). *n* = 6/group, except *n* = 4 in the atropine group.

To study whether generally used conduction system affecting drugs cause similar changes in C57Bl/6J mouse ECG as in human ECG, the effects of anticholinergic atropine (2 mg/kg ip), beta blocker metoprolol (2 mg/kg ip), and calcium‐channel blocker verapamil (5 mg/kg ip) were analyzed. Control saline ip injections increased HR at 15‐ and 20‐min timepoint (Fig. 3A). The largest HR increase of 70 bpm (18%) was seen 20 min after injections compared with baseline. There was a transient increase in PQ interval at 5 min, after which PQ interval returned to the normal level (Fig. 3E, M). EF increased 10–20 min after the injections from the baseline 59% to 72% at 20 min (Fig. 3I) most probably due to increased blood volume. Similarly than in the saline group, the HR was increased 15 and 20 min after the atropine treatment and the largest increase at 20 min was 63 bpm (16%) compared with baseline (Fig. 3B), meaning that atropine treatment itself did not increase the HR more than saline injections did. PQ interval began to get shorter at 10 min and was significantly shortened 15 and 20 min after atropine administration (Fig. 3F, N). We compared the baseline ECG with 15 and 20 min post atropine ECG by superimposing the ECG curves and noticed no flattening of the T‐wave shape (Fig. 3N). EF rose slowly and was significantly increased at 20 min (Fig. 3J).

Metoprolol decreased HR 5–15 min after the administration of the drug, and the largest decrease of 82 bpm (20%) was seen at 15‐min timepoint (Fig. 3C). PQ interval was increased 5–15 min after the metoprolol treatment (Fig. 3G, O) reaching highest effect at 10 min. EF was dropped to its smallest value at 5 min, after which it slowly rose being still significantly decreased at 15 min (Fig. 3K). Verapamil had similar effects than metoprolol, although its effect on HR was milder being 39 bpm (9%) at 10 min and shorter lasting from 5 to 10 min timepoint (Fig. 3D). Instead, the effect of verapamil on PQ interval was stronger, since it markedly increased PQ interval at 5–15 min (Fig. 3H, P). There was also a more pronounced drop in the EF at 5 min, after which EF started to return back to the normal level (Fig. 3L). No changes in other ECG parameters were seen after the saline, atropine, metoprolol, or verapamil administration, and there were no arrhythmias detected during the recordings (data not shown).

### The effects of acute myocardial infarction on ECG and echocardiography

**Figure 4:**
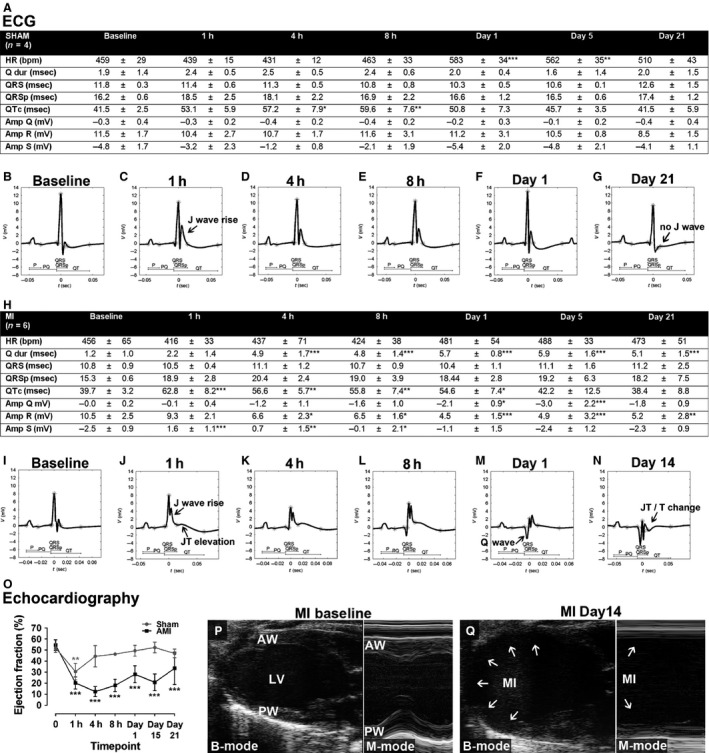
ECG and echocardiographic findings in acute myocardial infarction. ECG parameters 1 h to 21 days after AMI (H) and sham operation (A). Representative surface ECGs at different timepoints after AMI (I–N) and after sham operation (B–G). Ejection fraction (O) and echocardiographic images of anteroapical MI 14 days after LAD ligation (Q) in comparison to baseline (P). Results are expressed as mean ± SD. One‐way ANOVA with Dunnett's post hoc test was used, **P < 0.01, ***P < 0.001 compared with the baseline (0 min). *n* = 4 in the sham group and *n* = 6 in the MI group.

The early and late effects of AMI on ECG were studied at several timepoints (1 h to 21 days) after the induction of anteroapical infarction of the LV wall by LAD ligation and compared with sham operation. ECG results of the sham‐operated group showed a transient increase in QTc interval and HR (Fig. 4A) and a transient decrease in PQ interval at day 1 (data not shown). There was also a transient rise of the J wave within the first 8 h (Fig. 4B–E) seen also as a nonsignificant decrease in the S‐wave amplitude leaving the S amplitude negative (Fig. 4A). Although there was a rise in the J wave, the T wave kept its normal negative form (Fig. 4C–E). One day after the sham operation, the ECG was essentially similar to the baseline as well as at 21 days in half of the mice, and in the other half of the mice, the depression of the risen J wave had progressed to the point where no clear J wave was present (Fig. 4B, F, G). No changes were seen in the Q‐wave duration or amplitude (Fig. 4A) nor in the P‐wave duration or amplitude (data not shown).

After a large AMI, which affected most of the anterior and inferior wall of the LV (Fig. 4P, Q), there was a clear elevation in the JT segment at 1–8 h and the segment was still slightly elevated 1 day after the induction of AMI (Fig. 4I–M). JT elevation was associated with a rise of the J wave and a decrease in S amplitude within the first 8 h after AMI, which was greater compared with the sham group making the S‐wave amplitude positive 1–4 h after AMI (Fig. 4H–L). After the JT elevation became lowered, changes in JT/T segment were seen; the J wave got wider at day 5 and there was JT depression/T‐wave inversion starting at day 14 (Fig. 4N). The pathological Q waves appeared at 4 h when the duration of the Q wave was significantly increased lasting throughout the follow‐up. It was accompanied with a significant increase in Q‐wave amplitude at day 1 and day 5 (Fig. 4A, H–N). A decrease in R‐wave amplitude was seen at 4 h progressing toward day 1 after which it stayed at the same level through the follow‐up (Fig. 4H–N). There was a transient increase in QTc time seen, similarly to the sham‐treated group (Fig. 4H). No changes were seen in the P‐wave duration or amplitude or in the PQ time (data not shown). During ECG recordings, we did not observe arrhythmias, except for three mice which had premature ventricular contractions (PVCs) or premature atrial contractions (PACs) during one timepoint (1–2 PVCs at 8 h and day 14 and several PACs at day 14).

In echocardiography, the akinetic/hypokinetic area in the LV wall distally to the LAD ligation point could be clearly visualized already 1 h after AMI leading to marked decrease in EF measured with LV trace (Fig. 4O). The largest impairment in the systolic function was seen in the early phases within the first 8 h, after which the function improved somewhat toward the day 21 timepoint staying still markedly decreased. Also, in the sham group, a transient decrease of the EF was seen at 1 h due to global hypokinesia, but the systolic function recovered to the normal level already at 4 h. Permanent ligation of LAD led to a large anteroapical AMI affecting the ½–2/3 of LVAW, the inferior wall, and in some mice also the distal part of LVPW leading to thinning of the affected LV walls and to marked dilatation of LV already 14 days after AMI (Fig. 4P, Q). Histological findings of the infarcted hearts corresponded well with the echocardiography and showed areas of scar tissue corresponding to the akinetic/hypokinetic areas of LV. Small area of scar tissue in the LV wall was seen also in sham‐operated mice, which did not cause any visible changes in the echocardiography (data not shown).

### The effects of progressive LVH on ECG and echocardiography

**Figure 5:**
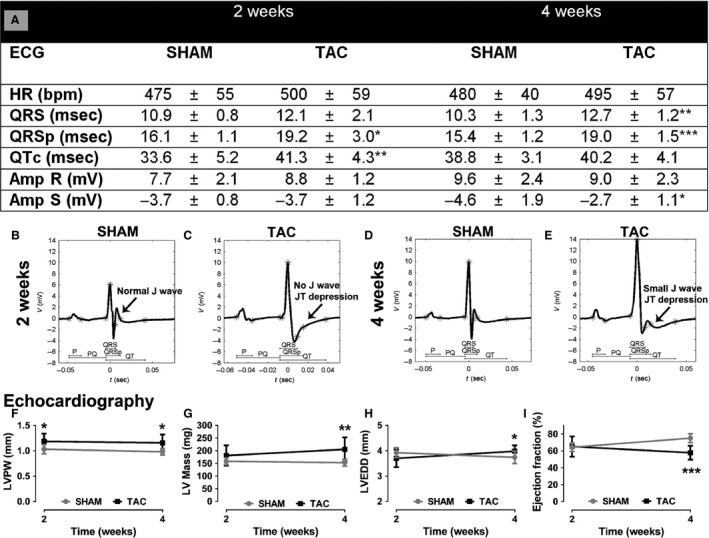
ECG and echocardiographic findings in progressive left ventricular hypertrophy at 2 and 4 weeks after the TAC operation. ECG measurements (A), representative surface ECGs of sham (B, D), and TAC operated (C, E) mice. Echocardiographic measurements of LVPW (F), LV mass (G), LVEDD (H), and ejection fraction (I). Results are expressed as mean ± SD. Student's paired *t*‐test was used to compare the sham‐ and TAC‐operated groups within a timepoint, *P < 0.05, **P < 0.01, ***P < 0.001. *n* = 8–10 mice/group.

The ECG and echocardiographic changes associated with progressive LVH were analyzed 2 and 4 weeks after cardiac pressure overload created by TAC operation. There was a tendency toward an increase in QRS complex width at 2 weeks (12%) and a significant increase at 4 weeks after TAC (24%). Even more marked changes were seen in QRSp duration, which was significantly increased already in the concentric phase at 2 weeks (19%) and also in the eccentric phase of LVH at 4 weeks (23%) (Fig. 5A–E). There were no significant changes in R‐wave amplitude; instead, the S‐wave amplitude was decreased 4 weeks after the TAC (Fig. 5A). QTc interval was increased at 2 weeks but not significantly at 4‐week timepoint. Repolarization abnormalities were seen in 44% of mice 2 weeks after TAC and in 40% of the mice 4 weeks after TAC. These changes included milder changes with J‐wave widening associated often with J wave lowering or more severe changes with a JT segment depression and disappearance/marked reduction of the J wave (Fig 5C, E). The ECG curves of the sham‐operated mice looked normal in shape (Fig. 5B, D). There were no differences in P‐ or Q‐wave duration, P‐ or Q‐wave amplitude, nor in PQ time between sham and TAC‐treated groups in either of the timepoints (data not shown). No arrhythmias were detected during the ECG recordings after the TAC operation.

The echocardiographic changes included a concentric hypertrophy phase at 2 weeks with increased LV wall thickness (Fig. 5F) and somewhat decreased LVEDD (Fig. 5H) leading to eccentric hypertrophy at 4 weeks after TAC with increased LV wall thickness (Fig. 5F), dilated LV (Fig. 5H), and decreased systolic function with EF of 58% compared with 75% in sham‐operated mice (Fig. 5I). The calculated LV mass increased 14% at 2 weeks and 34% at 4 weeks. No significant changes were detected in the left atrium area (data not shown).

## Discussion

Mouse models have become important with a large potential of studying molecular and genetic factors in cardiovascular diseases. However, there are challenges in reliable characterization of clinically relevant electrophysiological parameters in mice due to the small size, fast HR, and differences in surface ECG in comparison to man. Reliable and repeatable measurements of cardiac function are of great importance for characterizing mouse models and translating the results to human diseases. Therefore, we developed an accurate method for analyzing mouse ECG and validated it with the most widely used C57Bl/6J mouse strain. ECG findings and their correspondence with simultaneously recorded echocardiographic findings were studied in aging heart, after pharmacological manipulations with atropine, metoprolol, and verapamil, and in AMI and progressive LVH models.

Aging human heart is more prone to arrhythmias, such as atrial fibrillation, first‐degree atrioventricular block (PQ interval >0.20 sec), PACs, and PVCs. In addition, P‐wave duration, PQ interval time, and QRS complex width are increased, and QRS complex might be slightly aberrant and the amplitude slightly decreased (Fleg et al. 1990; Heikkilä and Mäkijärvi 2003; Kelley et al. 2006). It is not well known what ECG changes are caused by aging in mice, although some of them have been described earlier, namely an increased P‐wave duration and susceptibility to atrial fibrillation in Kunming mice (Luo et al. 2013) and increased PQ interval in C57Bl/6J mice (Chaves et al. 2003). Most of these findings were also seen in aging mouse heart in this study, as the prevalence of arrhythmias (PACs and PVCs), P‐wave duration, PQ interval, and QRSp duration were increased and R‐wave amplitude decreased. Also slight changes in QRS complex morphology were seen. The PQ intervals of healthy young mice were below 55 msec, and previously, it has been reported that the normal range of the PQ interval in mice is 30–56 msec (Kaese and Verheule 2012). Based on these findings, it could be suggested that the upper limit for normal PQ interval is 60 msec and PQ >60 msec equals for a first‐degree atrioventricular block in mice. With these criteria, the prevalence of the first‐degree atrioventricular block in C57Bl/6J mice in this study was 12% in the middle age group and 25% in the old mice group, which is comparable to old human populations describing the prevalence of 16–17% (Lakkireddy et al. 2003; Kelley et al. 2006).

In humans, it is hard to evaluate whether the ECG changes seen in old people are caused by normal aging process or whether they reflect an underlying subclinical heart disease (Lakkireddy et al. 2003). Wild‐type C57Bl/6 mice with normal chow‐diet do not develop atherosclerosis (Schreyer et al. 1998), and there were no signs of cardiac diseases based on echocardiography and macroscopical examination while they were killed. These differences between the species underline the importance of assessing the effects of aging on ECG in mice. ECG time intervals (RR, PQ, QRS, and QT) are also known to scale with body mass (Kaese and Verheule 2012), and therefore, also increased body mass of the older mice, and not the aging as such, might be the cause of the ECG changes seen during the aging in this study.

The correlation of increased LA size in echocardiography with increased P‐wave duration is fairly good in humans (Heikkilä and Mäkijärvi 2003), but to our knowledge, this has not been studied in mice, although an increased atrial size has been shown to accompany increased P‐wave duration in a transgenic mouse model (Rosenberg et al. 2012). Similarly than in humans, a significant correlation between LA size and P‐wave duration was seen. There were no correlations between LV mass and QRS duration, which would be expected based on the human studies (Heikkilä and Mäkijärvi 2003) and the TAC study herein indicating that the QRS widening in the TAC model is not only a result of increased LV mass. However, recently it has been shown that QRS duration in mice might underestimate the total ventricular activation time, and therefore, other methods might be needed for validating the QRS width (Boukens et al. 2013, 2014). We found a significant correlation between the LV mass and QRSp, the newly developed index, in normal C57Bl/6J mice. QRSp duration was possibly a more sensitive marker of increased LV mass also in the TAC experiment, since it was increased more pronouncedly already at 2 weeks compared with nonsignificant increase in QRS complex width (19% vs. 12%, respectively). Thus, an increase in QRSp duration might in fact be a more sensitive marker of LV mass increase than QRS complex width. This warrants further assessment of the QRSp changes in different LVH mouse models. PQ interval did not correlate with HR and P‐wave duration correlated, consistently with previous findings (Chaves et al. 2003).

Pharmacological manipulations with commonly used conduction system affecting drugs affected the cardiac electrophysiology as expected. Metoprolol and verapamil decreased the HR and increased the PQ interval, verapamil having a smaller effect on HR but stronger effect on PQ interval, as in humans (Koulu and Tuomisto 2007). In contrast to our findings, in C57Bl/6J mice, the verapamil treatment with twice as big dose as used here led to increase in HR together with an increase in PQ time (Speerschneider and Thomsen 2013). Atropine did not seem to increase the HR any further than the saline injections, but it decreased the atrioventricular conduction time as expected (Koulu and Tuomisto 2007). In fact in mice, sympathetic tone is predominant, in contrast to humans, and parasympathetic blockade (atropine) does not increase the HR and sympathetic blockade (metoprolol) causes a pronounced decrease in HR (Kaese and Verheule 2012), as seen in our study.

The ECG changes seen after AMI in C57Bl/6J mice included early JT elevation, marked reduction in S amplitude, pathological Q waves, decreased R‐wave amplitude, and later changes in JT/T segment. As far as is known, there are no studies describing thoroughly the mouse ECG changes after LAD ligation as shown in this study, but some of these changes have been described previously in CD2F1 mice (Wehrens et al. 2000) and in FVB mice (Gehrmann et al. 2001). In C57Bl/6J mice right after LAD ligation, a JT segment with positive large T wave was seen without clear ST/JT segment elevation (Speerschneider and Thomsen 2013), and it was interpreted that AMI in mice is rather reflected by S amplitude reduction and not ST elevation (Boukens et al. 2014). The S amplitude reduction was also seen in sham‐operated mice, although to a lesser extent, and therefore, the JT segment elevation was considered a more specific finding for early AMI in mice and corresponding to ST elevation in human. The JT segment change attenuated within a few days, and the LV dysfunction was greatest in the early phases of infarction, which is similar to humans (Heikkilä and Mäkijärvi 2003). The development of necrosis of myocardial cells in the ischemic area requires at least 2–4 h, and the entire process leading to an infarction scar takes at least 5–6 weeks to develop, during which the ECG findings change (Thygesen et al. 2012) as in our study as the JT elevation progressed in the early phase and Q‐wave duration and amplitude increased in the later phase after AMI.

To our knowledge, the surface ECG changes in the compensatory phase of LVH in the widely used TAC model have not been earlier characterized in mice to this extent. The QRS complex widening seen here is similar to human LVH (Iuliano et al. 2002; Thaler 2010) and has also been described in mice (Boulaksil et al. 2010). The increased LV mass in healthy old mice resulted only in very minor increase in QRS width; instead, in the LVH model with the similar size LV mass than in the old, the QRS was significantly prolonged. QRS width is affected both by heart size and impulse conduction velocity (Wiegerinck et al. 2006; Boulaksil et al. 2010), both of which may underlie the prolonged QRS since the LV mass was increased and the increased fibrosis associated to the TAC model (Huusko et al. 2012) may lead to increased conduction velocity. The primary diagnostic sign of LVH in humans is the increased QRS amplitude in the leads overlying the LV (I, aVL, V5‐V6) (Thaler 2010), which could not be detected herein, since the inferior lead II was recorded. The repolarization abnormalities seen here resembled the human secondary repolarization abnormalities, which are signs of severe LVH and reflect the onset of LV failure (Thaler 2010). Similar changes have also been described in the TAC model in the heart failure phase at 6–8 weeks in C57Bl/6J mice (Speerschneider and Thomsen 2013) and in a transgenic mouse model of massive concentric LVH (Sysa‐Shah et al. 2012).

The main limitation of this ECG analysis system is that we are recording just one lead representing the standard bipolar limb lead II, which views the heart at the frontal plane from the base toward the left leg and it most effectively views the inferior surface of the heart. In addition, although the analysis program itself is able to measure accurately HR, time intervals, and amplitudes, the analyzers themselves need to characterize the changes in the ECG waveform configurations, like JT/T changes and the nature of arrhythmias. The ECG recorded during isoflurane anesthesia is not fully equivalent with the ECG recordings of conscious mice due to effect on HR. Nevertheless, inhaled halogenated ethers like isoflurane are one of the anesthetics with the smallest effects on the mouse ECG, and in addition, the induction and recovery are quick, the depth of the anesthesia is easy to control, and it provides physiologically relevant HRs (Chaves et al. 2003). Recently, it was suggested that the HR correction for QT interval described by Mitchell et al. (Mitchell et al. 1998) is not recommended in anesthetized mice since repolarization duration is independent of HR, and instead, the absolute QT value should be preferred (Speerschneider and Thomsen 2013), but in this study, the QT and QTc values were comparable.

In summary, we have developed a novel ECG analysis algorithm for the analysis of mouse ECG. The electrocardiographic findings of aging mouse heart, after pharmacological manipulations and in AMI and progressive LVH models, resembled the changes seen in humans and also correlated well with the echocardiographic findings. This suggests that the ECG analysis algorithm developed herein is an accurate and reliable tool for analyzing mouse ECG noninvasively and together with the simultaneously recorded echocardiography provides thorough insights into mouse cardiac function in vivo.

## Conflict of Interest

None declared.
